# Genetic diversity and natural selection of *Plasmodium vivax* multi-drug resistant gene (*pvmdr1*) in Mesoamerica

**DOI:** 10.1186/s12936-017-1905-x

**Published:** 2017-07-01

**Authors:** Lilia González-Cerón, Alberto Montoya, Josselin C. Corzo-Gómez, Rene Cerritos, Frida Santillán, Marco A. Sandoval

**Affiliations:** 1 Centro Regional de Investigación en Salud Pública, Instituto Nacional de Salud Pública, Tapachula, Chiapas Mexico; 2 Departamento de Parasitología, Centro Nacional de Diagnóstico y Referencia, Ministerio de Salud, Managua, Nicaragua; 3 Division de Investigación, Facultad de Medicina, Universidad Nacional Autónoma de México, Mexico City, Mexico

**Keywords:** *Plasmodium vivax*, Southern Mexico, Nicaragua, *pvmdr1* polymorphism, Genetic diversity, Natural selection, Haplotype network

## Abstract

**Background:**

The *Plasmodium vivax* multidrug resistant 1 gene (*pvmdr1*) codes for a transmembrane protein of the parasite’s digestive vacuole. It is likely that the *pvmdr1* gene mutations occur at different sites by convergent evolution. In here, the genetic variation of *pvmdr1* at three sites of the Mesoamerican region was studied. Since 1950s, malarious patients of those areas have been treated only with chloroquine and primaquine.

**Methods:**

Blood samples from patients infected with *P. vivax* were obtained in southern Mexico (SMX), in the Northwest (NIC-NW) and in the northeast (NIC-NE) of Nicaragua. Genomic DNA was obtained and fragments of *pvmdr1* were amplified and sequenced. The nucleotide and amino acid changes as well as the haplotype frequency in *pvmdr1* were determined per strain and per geographic site. The sequences of *pvmdr1* obtained from the studied regions were compared with homologous sequences from the GenBank database to explore the *P. vivax* genetic structure.

**Results:**

In 141 parasites, eight nucleotide changes (two changes were synonymous and other six were nonsynonymous) were detected in 1536 bp. The PvMDR1 amino acid changes Y976F, F1076FL were predominant in endemic parasites from NIC-NE and outbreak parasites in NIC-NW but absent in SMX. Thirteen haplotypes were resolved, and found to be closely related, but their frequency at each geographic site was different (*P* *=* 0.0001). The *pvmdr1*_*codons* 925–1083_ gene fragment showed higher genetic and haplotype diversity in parasites from NIC-NE than the other areas outside Latin America. The haplotype networks suggested local diversification of *pvmdr1* and no significant departure from neutrality. The *F*_ST_ values were low to moderate regionally, but high between NIC-NE or NIC-NW and other regions inside and outside Latin America.

**Conclusions:**

The *pvmdr1* gene might have diversified recently at regional level. In the absence of significant natural, genetic drift might have caused differential *pvmdr1* haplotype frequencies at different geographic sites in Mesoamerica. A very recent expansion of divergent *pvmdr1* haplotypes in NIC-NE/NIC-NW produced high differentiation between these and parasites from other sites including SMX. These data are useful to set a baseline for epidemiological surveillance.

## Background

*Plasmodium vivax* causes most malaria cases in Latin America [1]. About 20 million people are at high risk and, in 2014, there were 390,000 confirmed malaria cases that resulted in 79 deaths [2]. From 2000 to 2015 (the end of the era of millennium development goals elaborated by the WHO), most countries in Mesoamerica (a region extending from central Mexico to Panama) reported a decline of at least 74% in the number of malaria cases [2]. In Nicaragua, still in the control phase, *P. vivax* and *Plasmodium falciparum* have been responsible for a fluctuating number of malaria cases during the last decade. From 2008 to 2011, there were less than 1000 cases reported, but the following years registered an increase in this number; in 2014, a total of 1163 cases were confirmed by microscopy. More than 70% of the cases reported in this country are produced in the Northeast or North Caribbean Coast Autonomous Region, while transmission is sporadic (with periodic outbreaks) in the Northwest side of the country. The most affected area of countries Nicaragua and Honduras is the indigenous Miskito region, which lies on both sides of the border (Northeast). Also in 2014, the number of reported malaria cases was 4931 in Honduras and 3380 in Guatemala [2], the latter country between Honduras and Mexico. Since 1999, only *P. vivax* has been transmitted in Mexico, with 696 cases reported in 2014. This country is in the pre-elimination phase, similar to El Salvador and Belize [2].

In Mesoamerica, two drugs have been used to treat malaria since the 1950s: chloroquine (CQ) and primaquine (PQ) [3]. CQ interferes with haemoglobin digestion by the parasite and causes death of asexual and sexual blood parasites. However, PQ is needed to eliminate dormant stages of *P. vivax* and young gametocytes of *P. falciparum* [4]. The first cases of *P. vivax* resistance to CQ were reported in Oceania and Asia, and later in South America [5]. The multidrug resistance 1 gene (*mdr1*) codes for a transport protein, which is comprised of two transmembrane domains and two ATP binding sites [6]. In Southeast Asia, a low in vitro sensitivity of *P. vivax* to CQ was associated with the PvMDR1 amino acid substitution Y976F [7, 8]. The gene fragment containing this and other polymorphisms (e.g. F1076L, also a transmembrane residue), have been found in distinct geographic sites and at different frequencies [9]. In South America, four haplotypes comprising residues (976/1076; Y/F, Y/L, F/F and F/L) were reported as well as one polymorphism (T958M) at a marginal transmembrane position [6, 7, 10–12]. In *P. vivax* from Honduras, the PvMDR1 haplotypes 976/1076; Y/L, F/F and F/L were described, but the wild type (Y/F; Sal I strain) was the most frequent (>70%) [13]. It is remarkable that in clinical studies at different geographic sites no association between PvMDR1 polymorphism and CQ resistance has been found [12, 14, 15]. Besides, a recent study suggested that *Pvmdr1* mutations emerged at different geographic sites possibly by convergent evolution [11].

Seeking to extend the genetic characterization of current *P. vivax* in Mesoamerica, parasites were obtained from three different geographic sites. Then, the occurrence of natural selection and the genetic relationships of *P. vivax* studying *mdr1* polymorphisms and haplotype frequency were investigated.

## Methods

### Blood samples and geographic origin

All infected blood samples were obtained from symptomatic patients seeking malaria diagnosis in Mexico and Nicaragua (in each case, after informed consent was given). From 2008 to 2010 in Mexico, 93 samples were obtained in the laboratory facility at the Regional Research Center for Public Health (CRISP-INSP) from patients living in the Tapachula municipality and its surroundings in southern Chiapas (SMX), the southernmost point of Mexico that borders with Guatemala [16]. From 2011 to 2012 in Nicaragua, 107 samples were selected from the sentinel laboratory network established by the Health Ministry at head municipalities, 83 from the Northeast (NIC-NE; RACCN, North Caribbean Coast Autonomous Region) and 24 from the Northwest (NIC-NW; North Pacific Coast). Another 14 samples were included that had been obtained in NIC-NW during 2006–2007 [17]. Distances between these sites are the following: ≈611 km from SMX to NIC-NW, ≈853 km from SMX to NIC-NE, and ≈331 km from NIC-NE to NIC-NW. The NIC-NE in comprised by the Miskito and the mining regions.

The diagnosis of *P. vivax* was carried out by microscopic examination of stained thick blood smears, coming from capillary blood samples (before treatment) used to impregnate filter paper (Whatman #2). Then the participants were administered the CQ-PQ combination treatment, which is in accordance with health standards in Mexico [18, 19] and in Nicaragua [20]. Genomic DNA was extracted from *P. vivax*-infected bloods impregnated on filter paper, using a commercially available QIAmp DNA blood Minikit (Qiagen, USA), and following the manufacturer’s instructions.

### PCR amplification and sequencing

A gene fragment of about 600 bp and containing codon positions 976 and 1076 was amplified using the following oligonucleotides: F3.2 5′˗ACC AGG ATA GTC ATG CCC˗3′ (nt 2747–2764) and R3 5′˗TCT CCC TTT AGG GAC ATC AAC˗3′ (nt 3384–3368). The PCR reaction was prepared as follows: 10 μL of 5× PCR buffer, 5 μL of MgCl_2_ (25 mM), 2.5 μL of the dNTPs (1.25 mM), 2.5 μL of each primer (10 μM), 0.5 μL of Go Taq DNA polymerase, 5 u/μL and 2–4 μL of sample DNA for a final volume of 50 μL. The PCR reaction conditions were as follows: 5 min at 94 °C followed by 35 cycles: 1 min at 94 °C, 1 min at 60 °C, and 1 min at 72 °C; afterwards, there was a final extension of 72 °C for 10 min.

To investigate the presence of other nucleotide changes reported previously [6], two additional nucleotide fragments were PCR amplified. To that, primers F1 5′˗GAG GGA GAT GTC ATC ATC AAC GA˗3′ (nt 1315–1337) and R1 5′˗CTT CTG TCC ACC TGA CAA CTT AG˗3′(nt 1733–1755), and F2 5′˗CAA GGA CAG CAA TGA GAA GAA˗3′ (nt 2013–2033) and R2 5′˗CGC GAT GAC TAA GAT GTA GAG G˗3′ (nt 2601–2622) were used. The PCR reaction was prepared as follows: 10 μL of 5× PCR buffer, 4 μL of MgCl_2_ (25 mM), 2.5 μL of dNTPs (1.25 mM), 1.9 μL of each primer (10 μM), 0.5 μL of Go Taq DNA polymerase 5 u/μL (Invitrogen Corporation, Carlsbad, CA), and 2–4 μL of extracted DNA for a final volume of 50 μL. The PCR reaction conditions were as follows: 3 min at 94 °C followed by 35 cycles: 40 s at 94 °C, 40 s at 54 °C, and 1 min at 72 °C; afterwards, there was a final extension of 72 °C for 5 min. All PCR reactions were run in a MyCycler (BioRad, Hercules, CA, USA).

All amplified products were resolved in agarose gels at 1%, and stained with 0.2 µg/mL ethidium bromide using an electrophoresis chamber Midicell primo (Thermo EC330, New York, USA). A molecular marker of 100 bp ladder was used (Invitrogen Corporation, Carlsbad, CA, USA). PCR products were then purified using a MiniElute PCR Purification Kit (Qiagen, Valencia, CA, USA), according to the manufacturer’s instructions. The purified products were Sanger sequenced by using forward and reverse primers (at the High Throughput Genomics Unit, Department of Genome Sciences, University of Washington, Seattle, WA, USA).

The quality of pherograms with the forward and reverse nucleotide sequences was verified manually and by using BioEdit v7.1.3 software. DNA sequences with rare polymorphisms were re-amplified and sequenced. All pherograms showed a single genotype pattern. The consensus sequences obtained for each gene fragment were submitted to the NCBI-Gen Bank [Accession Numbers: KX180164–KX180638].

### Data analysis

The Salvador I strain (Sal I) *mdr1*sequence was used as reference: XM_001613678. Sequences forward and reverse were aligned using ClustalW Multiple Alignment of BioEdit v7.0 [21] and revised manually. Nonsynonymous and synonymous changes were identified, and the frequency of each nucleotide change was calculated per geographic site in Mesoamerica (NIC-NE, NIC-NW and SMX), as well the haplotypes were constructed to background mutations at codons 976/1076 with the dnaSP program v5.1 [22]. Statistical analysis was carried out with STATA v12.1.

In order to explore the regional and global parasite relationships based on *pvmdr1*, haplotype networks were constructed using TCS 1.21 [23]. Mutational steps represent the connections between haplotypes, and empty squares showed the non-sampled or extinct haplotypes. The colour of the circles represents the geographic origins of each haplotype, while the size of the circle represents the frequency of each haplotype.

Indexes of nucleotide diversity were calculated in dnaSP v5.1. To test whether natural selection and gene flow shaped the evolution of *pvmdr1* gene in parasite populations of Mesoamerica, the number of synonymous (*s*) and nonsynonymous (*ns*) nucleotide changes and the difference in the rate of nonsynonymous versus synonymous changes (*dN˗dS*) were determined by using the Nei Gojobori proportion method with 1000 bootstrap replicates in MEGA v6.0 software [24]. Tajima’s D test and the minimal number of recombination events were calculated using dnaSP. To estimate the P*. vivax* genetic relationships from different locations, the *F*_ST_ values were estimated by using the two parameters of Kimura in dnaSP; values ranged from 0.0 to 1.0. A value of 0.0 indicates that populations are equal and 1.0 indicates they are completely different [25, 26].

DNA sequences for the global genetic analysis. Groups of DNA sequences that comprised codons 925–1083 (including positions 976/1076) were obtained from the NBCI Gene Bank. Brazil (BRZ): n = 7, EU333973-9 [5]; n = 3, AY571981-3 and 79–83, 85–89 [14]; n = 15, KM016495/502, 04, 05 and 07–17 [10]. Papua New Guinea (PNG): n = 6, AY571975-80 [14]. Iran (IR): n = 4, KM216181-4 (Sharifi-Sarasiabi et al. unpublished). Madagascar (MD): n = 80, EU683813-19 [14]. South Korea (SK): GU476519 (Chen et al. unpublished). India (IND): n = 49, KC818349-78, 80, 82, 85, 87, 89, 90, 92, 93, 95, 96, 98, 99/400, 02, 04, 06–09, 11 and 12 [27]. Cameroon: n = 8, KJ534638-45 (Ngasa et al., pers.comm.). Additionally, other DNA sequences were reconstructed, using the aforementioned sequence reference of the Sal I strain as a template (AY618622, with codon 958 atg/M; AY571984 and XM001613678 with codon 958 acg/T), and only if the length and coordinates of the *pvmdr1* gene fragment, synonymous and nonsynonymous nucleotide changes, and haplotype frequency were clearly described: IND, n = 25 [28]. Honduras (HON), n = 37 [13]. Nepal (NEP), n = 39 [29]. Ecuador (ECU), n = 17; Sri Lanka (SLK), n = 119; Pakistan (PK), n = 24; Sudan (SUD), n = 4; Sao Tomé (SAT), n = 3 [11].

## Results

### *Pvmdr1* gene polymorphism

Upon analysing 1536 bp of *pvmdr1*, two synonymous and six nonsynonymous nucleotide changes were detected (Table 1). From 163 parasites (66, 23 and 74 from NIC-NE, NIC-NW and SMX, respectively), two changes (one nonsynonymous and one synonymous) were detected in the gene fragment of 411 bp (nucleotides 1330–1740). The nonsynonymous change was observed (only in NIC-NW) at codon 500 D → N). While the synonymous change at codon 529, was highly frequent in SMX and NIC-NE, but not detected in parasites from NIC-NW. Interestingly, from 155 parasites (65, 26 and 64 from NIC-NE, NIC-NW and SMX, respectively) a second fragment of 549 bp (nucleotides 2767–3315) was obtained, and six nucleotide changes were detected. The nonsynonymous changes at codons Y976F and F1076L were highly frequent in NIC-NE and NIC-NW, but not detected in SMX. Similarly, the synonymous change at codon 1021 was highly frequent in NIC-NE and NIC-NW, but rare in SMX. The change at codon A927T exclusive to NIC-NE, it was detected in parasites from different municipalities of the Miskito and the mining regions. The substitution T958M was highly frequent at the three sites, while the F1070L change was detected in only two isolates from NIC-NE (Rosita and Prinzapolka municipalities) and two isolates from SMX (from the year 2010). The third gene fragment of 576 bp (nucleotides 2032–2607), obtained from 148 parasites (63, 17 and 72 from NIC-NE, NIC-NW and SMX, respectively), was identical to the Sal I strain sequence.

**Table 1:** Gene and protein polymorphism of *Plasmodium vivax* MDR1 from different sites in Mesoamerica

Gene fragment (codons)	444–580 _nt 1330–1740_			923–1105 _nt 2767–3315_						
	(Codon number) codon/one letter amino acid code									
Codon number		(500)	(529)		(927)	(958)	(976)	(1021)	(1070)	(1076)
Sal I sequence^a^		gat/D	aca/T		gca/A	acg/T	tac/Y	ttc/F	ttc/F	ttt/F
		a../N	..g/T		a../T	.t./M	.t./F	..t/F	c../L	c../L
	*n*			*n*						
	Frequency (%) of the nucleotide changes									
Southern Mexico, 2008–11	*74*	0	97.3	*64*	0	96.8	0	4.6^b^	3.2^b^	0
Nicaragua										
NE, 2011–12	*66*	0	59.1	*65*	24.6	95.4	63.1	93.8	3.1^c^	86.1
NW, 2012	*16*	100	0	*18*	0	100	100	100	0	100
NW, 2006–7	*7*	28.5	57.1	*8*	0	87.5	62.5	37.5	0	62.5
Total samples	*163*			*155*						

*SMX* southern Mexico, *NE* Northeast Nicaragua, *NW* Northwest Nicaragua, *n* number of isolates, *D* aspartic acid, *T* threonine, *A* alanine, *Y* tyrosine, *F* phenylalanine, *N* asparagine, *M* methionine, *L* leucine

^a^Sal I strain sequence; XM001613678

^b^Mutation detected in 2010 samples

^c^Three samples had a nonsynonymous mutation (Rosita and Prinzapolka)

An additional *pvmdr1* fragment of 576 bp (codons 678–869) was identical to Sal I from samples obtained at NE (n = 63), NW (n = 17) in Nicaragua and SMX (n = 73), respectively

### *Pvmdr1* haplotypes and its geographic frequency

**Fig. 1:**
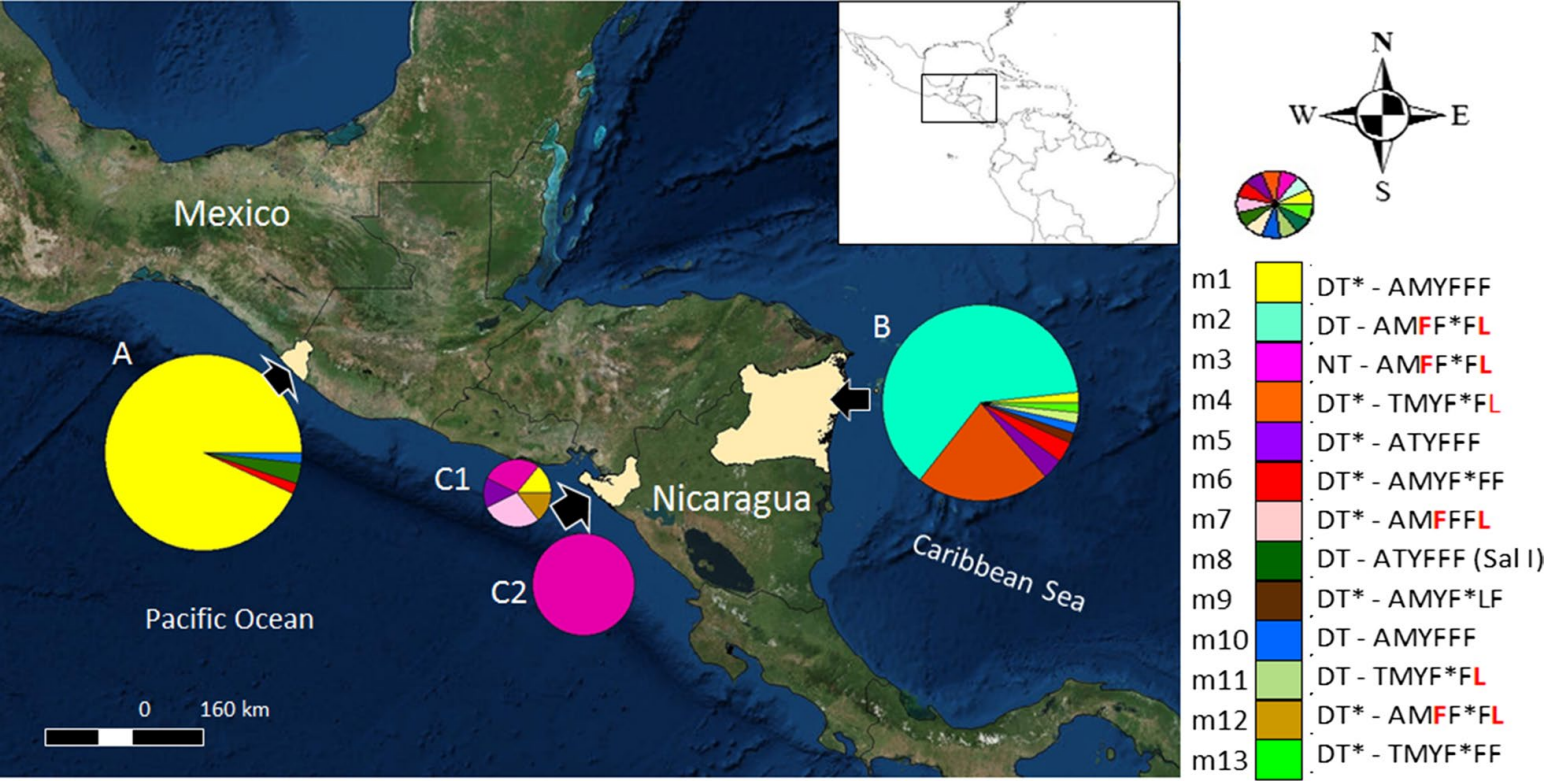
Geographic distribution of the *P. vivax mdr1* haplotypes in Mesoamerica. Gene polymorphism was detected at codons 500, 529–927, 958, 976, 1021, 1070 and 1076. It includes 141 isolates (59, 59 and 23 from SMX, NIC-NE and NIC-NW, respectively). (*A* n = 59) SMX and (*B* n = 59) NIC-NE. In NIC-NW, six haplotypes were detected in 2006–2007 (*C1* n = 7), while only one was detected in 2012 (*C2* n = 16) (*Asterisk* synonymous changes). The *circle* indicates *pvmdr1* haplotypes (n = 13; by colours); Sal I; *pvmdr1*, XM001613678*. One*-*letter code* indicates amino acid: *D* aspartic acid; *T* threonine; *A* alanine; *Y* tyrosine; *F* phenylalanine; *N* asparagine; *M* methionine; *L* leucine. The amino acid changes at codons 976 and 1096 are shown in *red*. *n* number of samples

There were 141 parasite isolates (59 from NIC-NE, 59 from SMX and 23 from NIC-NW) with the nucleotide information at variant codons 500, 529–927, 958, 976, 1021, 1070 and 1076 resolved 13 *pvmdr1* haplotypes (m1 → m13; ordered by their frequency) (Fig. 1). There were differences in haplotype frequencies between the three sites (*P* = 0.000): haplotype m1 GG-GTACTT was predominant (93.2%) in SMX; m3 AA-GTTTTC was highly frequent (78.2%) in NIC-NW; m2 GA-GTTTTC and m4 GG-ATATTC were at 62.7 and 22%, respectively, in NIC-NE. Other haplotypes were detected in two or all sites, although at a very low frequency. For instance, haplotype m1 was also detected in one isolate from NIC-NE and in another from NIC-NW, m6 and m10 were observed at SMX and NIC-NE, and m5 was found in NIC-NW and NIC-NE. One haplotype (m8) was exclusive to SMX and resembled the Sal I strain sequence. Another three haplotypes (m9, m11 and m13) were exclusive to NIC-NE and two (m7 and m12) to NIC-NW (Fig. 1). There were four haplotypes having nucleotide changes at codons 976F (a → t) and 1076L (t → c); m2, m3, m7: GG-GTTCTC, m12: GG-GTTTTC; those haplotypes differed among them by one to three mutations. Two other haplotypes showed only 1076F/L; m4 and m11: GA-ATATTC. The haplotypes m2 and m4, highly frequent at NIC-NE were also frequent in all sampled municipalities: Rosita, Bonanza, Waspam and Puerto Cabezas. The higher number of exclusive haplotypes were detected in Waspam (n = 3), the Miskito region that borders Honduras.

### Genetic relationship of *pvmdr1* haplotypes from SMX, NIC-NW and NIC-NE

**Fig. 2:**
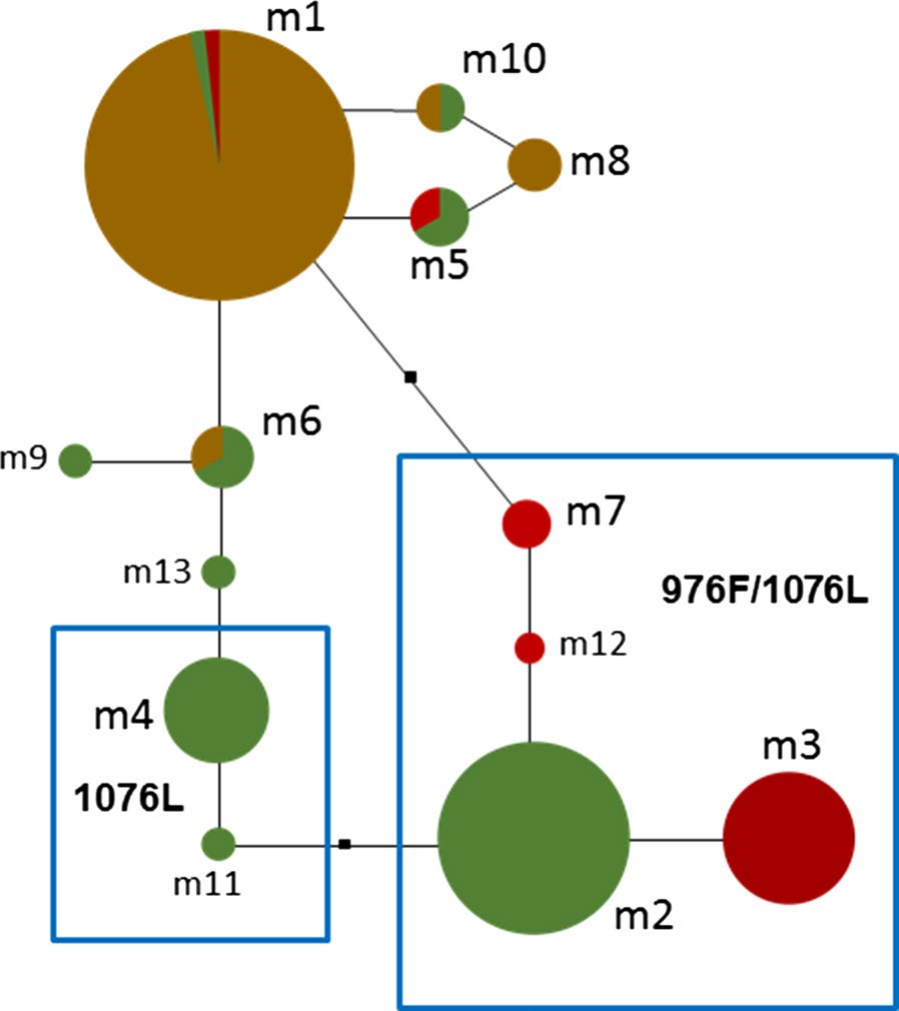
Haplotype network of the *P. vivax mdr1* from the study region. The network includes haplotypes from Southern Mexico (*light brown*), Northeast (*green*) and Northwest (*dark red*), Nicaragua. *Each circle* indicates a different haplotype, and lines are connecting them. The *black square* indicates a haplotype not sampled or extinct. All haplotypes were closely related and signs of gene diversification were detected. Sal I haplotype: XM001613678 correspond to haplotype m8. The network includes 141 sequences. The haplotypes showing 1076L and 976F/1076L are within a *blue frame*, respectively

The haplotype network of *P. vivax mdr1* comprising mutations at codons 500, 529–927, 958, 976, 1021, 1070 and 1076 for parasites from the study sites is shown in Fig. 2. All 13 haplotypes were found closely related; most haplotypes were at one mutational step from each other, and at 1–9 mutational steps among them. Haplotypes from SMX were only separated by 1–2 mutational among them. The most frequent haplotype (m1) in SMX was at one mutational step from the Sal I strain sequence. While haplotypes m2 and m3 were at about 6–9 mutational steps from m1; they were highly frequent in NIC-NE and NIC-NW, respectively.

### Genetic diversity

Parasites from NIC-NE and mostly from the Mesoamerican region showed a nucleotide diversity (π = 0.0036) similar to that found at other geographic origins as well as the global level (π = 0.0028). Moreover, the minimal number of recombination events were from very low to cero (the value for Mesoamerica was Rm = 2) (Table 2).

**Table 2:** Genetic parameters of *P. vivax mdr1* gene in Mesoamerica and other geographic sites

Genetic parameters	Mesoamerica						South America		Outside America					
	Total	SMX	NIC-NE	NIC-NW	HON	MA	BRZ	ECU	IR_PK	IND	SLK	NEP	AFR	MD
N	602	64	65	26	37	192	38	17	28	75	119	39	15	80
SS and M	18	3	6	4	2	6	8	1	3	3	2	5	4	1
H	19	4	7	4	4	10	7	2	4	4	3	5	4	2
Hd	0.769	0.150	0.553	0.348	0.377	0.666	0.414	0.441	0.206	0.462	0.533	0.398	0.638	0.025
π	0.0027	0.0004	0.0028	0.0017	0.0011	0.0036	0.0016	0.0009	0.0006	0.0012	0.0012	0.0010	0.0018	0.00005
θ	0.0054	0.0013	0.0026	0.0022	0.001	0.0022	0.0039	0.0006	0.0016	0.0009	0.0008	0.0024	0.0025	0.0004
Rm	2	0	1	0	1	2	1	0	0	0	0	0	0	0
Tests of neutrality														
Tajima’s D	−1.161	−1.297	0.181	−0.5699	0.3077	1.361	−1.681	0.949	−1.527	0.7437	0.853	−1.513	−0.922	−1.0526
*dN*–*dS*: Z value/*P*	0.116/0.908	−0.598/0.551	1.108/0.270	−0.534/0.595	1.44/0.153	−0.295/0.769	−0.570/0.57	1.046/0.298	1.667/0.098	1.337/0.184	1.371/0.173	1.845/0.067	0.416/0.678	1.047/0.297

The fragment comprising 477 bp was analyzed (nucleotide fragment 2773–3249); Z test of selection (HA: dN=/=dS)

*SMX* southern Mexico, *NIC*-*NE* Northeast Nicaragua, *NIC*-*NW* Northwest Nicaragua, *HON* Honduras, *BRZ* Brazil, *ECU* Ecuador, *IR*_*PK* Iran and Pakistan, *IND* India, *SLK* Sri Lanka, *NEP* Nepal, *AFR* Africa (includes samples from Sudan (n = 4), Cameroon (n = 8) and São Tomé (n = 3), *MD* Madagascar, *N* number of sequences, *SS* number of segregating sites, *M* nucleotide substitutions, *H* number of haplotypes, *Hd* haplotype diversity, *π*/*θ* indexes of diversity, *Rm* minimal number of recombination events

### Natural selection

Tajima’s D test statistical value was negative for SMX and NIC-NW, while being positive for NIC-NE and other sites, but no significant. The lowest value was for Brazil (−1.681) and the highest for Ecuador (0.949), but in either case, it was found to be no significant. Although a higher number of nonsynonymous than synonymous changes were detected, the *dN*–*dS* values showed no significant departure from neutrality. Parasites from Nepal had the highest *dN*–*dS* value with marginal significance (1.845; *P* = 0.067) (Table 2).

### Haplotype relationships

**Fig. 3:**
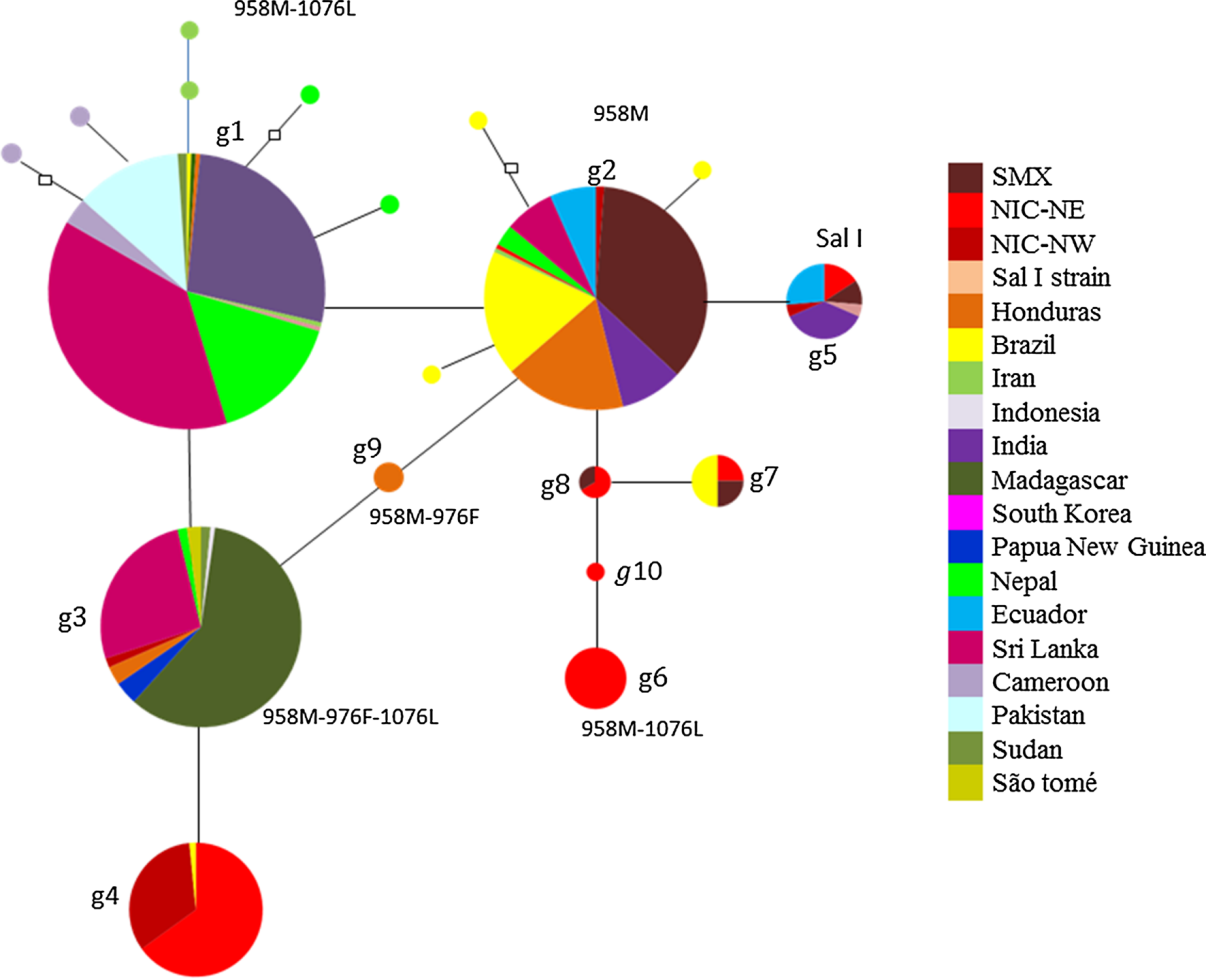
Haplotype network of *P. vivax mdr1* gene from different geographic origins. The network includes 610 sequences and 19 haplotypes. Parasites from Northeast Nicaragua (NIC-NE) and Brazil had the highest number of haplotypes (n = 7). *Each circle* indicates a different haplotype (n = 19) connected by lines. The *empty squares* indicate haplotypes not sampled or extinct. *Each colour* indicates a different country. Three frequent haplotypes were detected at different geographic sites, respectively. One haplotype was frequent in Latin America. Signs of gene diversification were found at the regional and at local level. Sal I haplotype: XM001613678. A gene fragment of 479 bp (nt 2772–3250) was used. NIC-NW, Northwest, Nicaragua. SMX, Southern Mexico

When using global sequences of *P. vivax mdr1*, the haplotype network evidenced the relationship of 19 global haplotypes (g1 → g19) identified in 610 sequences (Fig. 3). The most frequent haplotype g1 and probably the ancestral haplotype, it was predominant in parasites from Sri Lanka, India, Nepal, Pakistan and Madagascar and existed in few parasites from Latin America. In contrast, the haplotype g2 was highly frequent in parasites from Latin America (SMX, Brazil, Honduras and Ecuador). Haplotype g2 was at one mutational step from g5, the latter represent Sal I strain (the only haplotype with codon 958 → acg) which was found in parasites from Latin America and India. Moreover, from g2 emerged a branch of four descendant haplotypes (restricted to American parasites), one present in SMX and NIC-NE (connected to another haplotype present in parasites from SMX, NIC-NE and Brazil), and two exclusive to NIC-NE (Fig. 3). Whereas, haplotype g3 was included in one parasite from Brazil and another from NIC-NW, is at one mutational step from g4, the latter of which showed high frequency in NIC-NE and NIC-NW parasites and was found in one parasite from Brazil (Fig. 3). There were two different haplotypes (g1 and g6) showing polymorphisms T958M and F1076L. Haplotype g6 exclusive in NIC-NE emerged from a different branch of that detected in one parasite from Brazil and other geographic sites outside Latin America (g1).

### *F*_ST_ values of *P. vivax* between Mesoamerica and other geographic origins

*F*_ST_ values were mostly from low to moderate between sites inside Latin America, excluding NIC-NE and NIC-NW. *F*_ST_ values, between SMX, Brazil, Honduras and Ecuador ranged from 0.015 to 0.194. The lowest value was between SMX and Brazil, and the highest between Honduras and Ecuador. In Asia (mostly the Indian subcontinent: Nepal, Iran, Pakistan, India and Sri Lanka), *F*_ST_ values were from low to moderate (Table 3). Between NIC-NE and NIC-NW the value was also low (0.097), but higher between these sites and others inside and outside Latin America (0.4784–0.8039). In general, between Latin American and other sites outside this continent, *F*_ST_ values were generally high (0.3813–0.9430).

**Table 3:** F_*ST*_ values of *P. vivax*, using the *mdr1* gene fragment, within Mesoamerica and between different geographic sites

	SMX	NIC-NE	NIC-NW	HON	BRZ	MD	IND	SLK	ECU	IR-PK	AFR
NIC-NE	0.716										
NIC-NW	0.803	0.097									
HON	0.113	0.632	0.708								
BRZ	0.015	0.615	0.698	0.064							
MD	0.943	0.609	0.609	0.825	0.818						
IND	0.549	0.580	0.671	0.381	0.392	0.774					
SLK	0.694	0.518	0.582	0.511	0.543	0.613	0.180				
ECU	0.149	0.701	0.780	0.194	0.136	0.897	0.498	0.655			
IR-PK	0.790	0.621	0.721	0.631	0.613	0.864	0.145	0.169	0.735		
AFR	0.668	0.478	0.524	0.510	0.544	0.477	0.203	−0.011	0.639	0.144	
NEP	0.685	0.578	0.670	0.514	0.518	0.777	0.055	0.090	0.642	0.021	0.100

The fragment comprising 477 bp was analysed (nt 2773–3249)

*SMX* southern Mexico, *NIC*-*NE* Northeast Nicaragua, *NIC*-*NW* Northwest Nicaragua, *HON* Honduras, *BRZ* Brazil, *ECU* Ecuador, *IR*_*PK* Iran and Pakistan, *IND* India, *SLK* Sri Lanka, *NEP* Nepal, *AFR* Africa, which includes Sudan (n = 4), Cameroon (n = 8) and São Tomé (n = 3), *MD* Madagascar

## Discussion

The Mesoamerican region has ecological and demographic conditions that favor malaria transmission. Chloroquine (CQ) and primaquine (PQ) have been the only drugs used in this region to treat patients infected with *P. vivax* and/or *P. falciparum* [19, 20, 30] and, so far, both *Plasmodium* species seem susceptible to CQ. According to this, mutations at *pvmdr1* might not be associated to CQ resistance. Therefore, PvMDR1 976F and CQ resistance in vitro seemed to be a counterfeit association [8]. In fact, clinical studies have shown that polymorphisms at *pvmdr1* e.g. 976F and others adjacent are not associated with CQ resistance [12, 14, 28], and there are no functional studies exposing *pvmdr1* mutations participating in the development of CQ resistance. Schousboe et al. [11] suggested that *pvmdr1* mutations 958M, 976F and 1076L arose independently and several times at different geographic sites. In Mesoamerica, the selective force of natural selection on *pvmdr1* differed spatially, with lack of statistical significance. Besides, recent genomic studies have not revealed signature of selection on *pvmdr1* in parasites from different geographic sites [31–33], including parasites from the Southeast Asia [33] where high levels of CQ resistance have been reported [8, 34].

Malaria has been historically hypo endemic in Mesoamerica. From 2000 to 2010 a reduction in more than 75% was recorded in several countries including Nicaragua and Mexico. Contrarily from 2012 to 2014, Nicaragua was the only country in Mesoamerica that reported a significant increase in malaria cases [2]. In this country, while intense and effective control strategies caused reduction in the number of malaria cases, climate changes and delay in the delivery of control measures have probably caused the increase in malaria transmission. Alike southern Mexico, in NIC-NW and NIC-NE several haplotypes exposing changes 976F and/or 1076L were exclusive and highly frequent. The haplotype networks suggest that these mutations resulted from a recent diversification in Mesoamerica. It is remarkable that haplotypes expressing 1076L, present in parasites from NIC-NE (haplotype g6) seemed the result of different evolutionary pathway than in other geographic sites (haplotype g1).

The presence of some *pvmdr1* haplotypes in all sites (SMX, NE-NIC and NW-NIC), may be attributed to ancient parasite flow across the Mesoamerican region. It was notorious a predominance of blood infections with a single *pvmdr1* haplotype, presumably monoclonal infections. A clonal transmission of *P. vivax* might be occurring in the region. It is supported by the lack of mixed genotype infections for *pvmsp1*_42_ a gene fragment, of moderate diversity, reported in NIC-NE/NW [35]. In SMX, the rate of mixed genotype infections has been negligible during the last decade [16, 36].

In Mesoamerica, parasite flow might be controlled by different factors, including separation by distance, geographic barriers, cultural and language barriers, poor road infrastructure, the effectiveness of the malaria control interventions. There is a volcanic arc in between NIC-NE and NIC-NW. At either side of the arc exclusive *pvmdr1* haplotypes were detected. Similarly, for the blood stage antigen *pvmsp1*_*42*_ a divergent haplotype was exclusive in Chinandega municipality (NIC-NW) [35]. In NIC-NE, the most frequent *pvmdr1* haplotypes in the region, so were at the municipality level. A similar spatial pattern of haplotypes was reported for *pvmsp1*_42_ [35]. Yet, the presence of exclusive haplotypes suggests partial parasite flow between the Miskito and mining regions in NIC-NE. By using microsatellites, the analysis of *P. falciparum* exposed that in the mining region there was a genetic population distinct from that detected in the Miskito area [37]. The Miskito ethnic group (Waspam and Puerto Cabezas) live in multiple rural communities and it is partly inaccessible due to cultural and language barriers. Alike *P. falciparum*, *P. vivax* cause relapse episodes of long latency, an additional risk of parasite dispersion [38], and microsatellite markers provided higher resolution than single nucleotide polymorphism, and subtle genetic changes can be detected [39]. It is probable that some *pvmdr1* mutations might have appeared at different sites in Mesoamerica as formerly suggested for other geographic sites [11].

The results from this study and the epidemiological and demographic scenario suggest that a strong genetic drift might have influenced the spatial differences of the *pvmdr1* haplotype frequencies in Mesoamerica. The Sal I type was only detected in SMX and at low frequency. In Honduras, parasites isolated from 2004 to 2009 exhibited haplotypes with PvMDR1 976F and/or 1076L changes at a frequency of <30% [13]; while in the Northeast Nicaragua area the frequency of those changes was almost 90%. These changes were also present in strains causing an outbreak in the Northwest Nicaragua in 2012, it corresponded to an exclusive haplotype. In South America, mutations 976F and 1076L were reported to have low frequency [12, 31].

*Plasmodium vivax* arriving at the American continent faced adaptive processes triggered by different local factors e.g. the specificity of the *Anopheline* species, the host immune pressure, the anti-malarial drugs employed, and the ecological conditions to mention some reasons [40–42]. Genomic studies have exposed that *P. vivax* exhibits low diversity and high differentiation in Latin America [42, 43]. Analysis of the mitochondrial genome exposed low *F*_ST_ values between South America and Central America, the last ones corresponded to ten strains obtained in the 1970s [44]. Evidence based on the entire *pvmdr1* gene also supports a high genetic differentiation between old and new world parasites [32]. In the present study, the *F*_ST_ value using the *pvmdr1*_codons925–1083_ matched partially the previous results, also suggesting a strong differentiation globally but low-moderate differentiation at regional level. Excepting parasites from NIC-NE/NW, these were highly differentiated from all other parasites including those from SMX and Honduras, possibly given by the recent expansion of *pvmdr1* divergent haplotypes.

Regardless of its origin, *P. vivax* has been at some extent circulating in the American continent. Southern Mexico is a transit area for human migrations coming from South and Central America [45], and still a proportion of humans migrants entering North America can be potentially infected with *P. vivax* [46, 47]. Therefore, further genomic studies are necessary to uncover the evolution of *P. vivax* in the Mesoamerican region comparing to other sites of America and worldwide. Furthermore, molecular monitoring of *P. vivax mdr1* at different sites in Mesoamerica would help to understand transmission dynamics. Because of its variability and spatial patterns, *pvmdr1* is an important marker for epidemiological surveillance to support the current initiatives to control and eliminate malaria from Mesoamerica.

## Conclusions

These results suggest a recent diversification of *pvmdr1*which likely occurred at a regional level, natural selection varied spatially and was mostly in a weakly to-mild manner. In this hypo-endemic region, a strong genetic drift might have caused the great differences in *pvmdr1* haplotype frequency among the studied sites, and hence the high differentiation of parasites between NIC-NW/NIC-NE and SMX or other sites. These data are relevant to set a baseline level for epidemiological surveillance in the region.

## Abbreviations

*Pvmdr1*: *P. vivax* multidrug resistance 1 gene
CQ: chloroquine
PQ: primaquine
SMX: southern Chiapas, Mexico
NIC-NE: Northeast, Nicaragua (it is part of the North Caribbean Coast Autonomous Region)
NIC-NW: Northwest, Nicaragua (North Pacific Coast)
